# The impact of *FOXP3* polymorphisms on oral cancer progression and clinicopathological characteristics

**DOI:** 10.7150/jca.84470

**Published:** 2023-05-05

**Authors:** Ping-Ju Chen, Chiao-Wen Lin, Hsueh-Ju Lu, Chun-Yi Chuang, Shun-Fa Yang, Ying-Erh Chou

**Affiliations:** 1 Institute of Medicine, Chung Shan Medical University, Taichung, Taiwan; 2 Department of Dentistry, Changhua Christian Hospital, Changhua, Taiwan; 3 Department of Post-Baccalaureate Medicine, College of Medicine, National Chung Hsing University, Taichung, Taiwan.; 4 Institute of Oral Sciences, Chung Shan Medical University, Taichung, Taiwan; 5 Department of Dentistry, Chung Shan Medical University Hospital, Taichung, Taiwan; 6 Division of Hematology and Oncology, Department of Internal Medicine, Chung Shan Medical University Hospital, Taichung, Taiwan; 7 School of Medicine, Chung Shan Medical University, Taichung, Taiwan; 8 Department of Otolaryngology, Chung Shan Medical University Hospital, Taichung, Taiwan; 9 Department of Medical Research, Chung Shan Medical University Hospital, Taichung, Taiwan

**Keywords:** oral cancer, FOXP3, polymorphism, betel quid

## Abstract

Oral cancer is the sixth leading cause of cancer mortality worldwide. Genetic, epigenetic, and epidemiological risk factors were suggested to be correlated with the carcinogenesis of oral cancer. In this study, we focused on the correlations of *FOXP3* single-nucleotide polymorphisms (SNPs) to oral cancer susceptibility and clinicopathological characteristics. The *FOXP3* SNPs rs3761547, rs3761548, rs3761549, and rs2232365 in 1053 controls and 1175 male patients with oral cancer were analyzed with real-time polymerase chain reaction. The results showed that the betel quid chewer who carried the *FOXP3* rs3761548 polymorphic variant “T” were significantly associated with lower risk to develop oral cancer [AOR (95% CI) = 0.649 (0.437-0.964); p = 0.032]. The betel quid chewers with genotypic variant “T” of *FOXP3* rs3761548 in male oral cancer patients were associated with lower risk of cell differentiated grade [AOR (95% CI) = 0.592 (0.377-0.930); p = 0.023]. The carriers of *FOXP3* rs3761548 polymorphic variant “T” in male oral cancer patients with alcohol consumption were associated with lower risk to develop greater tumor [AOR (95% CI) = 0.609 (0.378-0.983); p = 0.042] and lower risk of cell differentiated grade [AOR (95% CI) = 0.440 (0.248-0.779); p = 0.005]. In conclusion, our results have revealed that the *FOXP3* rs3761548 polymorphic variant “T” was associated with lower risk of oral cancer susceptibility, greater tumor size, and cell differentiated grade among betel quid chewers. The *FOXP3* rs3761548 polymorphisms may play a role as pivotal biomarkers to predict oral cancer disease development and prognosis.

## Introduction

Oral cancer is the sixth leading cause of cancer mortality worldwide 1, 2. Genetic, epigenetic, and epidemiological risk factors such as tobacco smoking, alcohol consumption, betel quid chewing, ethnicity, familial and genetic predisposition, immunosuppression, viruses, and radiation were suggested to be correlated with the carcinogenesis of oral cancer 3-7. Transcription factor forkhead box protein 3 (FOXP3) is a member of the family of forkhead transcription factors and is mainly localized in the nucleus 8. The FOXP3 was suggested as the main marker of regulatory T cells (Tregs) 8, 9. It was suggested that the Treg cell differentiation and function was depended on FOXP3 expression 8-11, and the Tregs were suggested to play a critical role in oral squamous cell carcinoms (OSCC) 12, 13. The tumor microenvironment (TME) is a complicate system composed by cancer cells, immune cells and stromal cell 8, 14, 15. Tregs in the TME were suggested as checkpoint which blockade immune surveillance of tumors and suppress antitumor immune responses 8, 14, 16.

Previous studies have suggested that the tumor-infiltrating FOXP3 positive T cells were associated with development and poor prognosis in OSCC 13, 17, and the *FOXP3* polymorphisms were associated with cancer development and prognosis in various cancers such as cervical cancer 18, 19, gastric adenocarcinoma 20, and colorectal cancer 21. However, the associations and influences of *FOXP3* polymorphisms to OSCC tumor progression and clinicopathological characteristics remained not well-investigated. In this study, we focused on four SNPs of *FOXP3* rs3761547, rs3761548, rs3761549, and rs2232365, and try to elucidate their associations to oral cancer susceptibility and clinicopathological characteristics with environmental risk factors.

## Materials and Methods

### Study subjects

A total of 1175 male oral cancer patients and 1053 cancer-free controls were enrolled in our study. All the participants were recruited during 2016 to 2020 at Chung Shan Medical University Hospital in Taichung, Taiwan. For the TNM staging, the oral cancer patients who enrolled in our study were staged clinically at the time of diagnosis according to the American Joint Committee on Cancer (AJCC). The tumor differentiation was examined and rated according to the AJCC classification by pathologist. The demographic data such as age and gender were reported by each participant. Individuals who without oral precancerous disease, including leukoplakia, erythroplakia, and history of cancer of any sites were enrolled as the control group of our study. This project was approved by the institutional review board of Chung Shan Medical University Hospital (IRB number: CS1-21151), and informed written consent was provided to each patient who enrolled in this study.

### Sample preparation and DNA extraction

The peripheral blood specimens from oral cancer patients and normal controls who participated in our study were collected for genomic DNA extraction 22. The genomic DNA extraction assay was performed following the manufacturer's manual of QIAamp DNA blood mini kits to acquire the DNA. The final step of DNA elution was completed with the Tris-EDTA (TE) buffer. The extracted DNA was used as DNA template in further real-time polymerase chain reactions (PCRs) 23.

### Selection of *FOXP3* SNPs

In the current study, a total of four *FOXP3* SNPs rs3761547, rs3761548, rs3761549, and rs2232365 were selected based on the International HapMap Project database 24. The *FOXP3* rs3761547 was selected because the *FOXP3* rs3761547 polymorphisms was suggested as a male-specific risk factor in multiple sclerosis 25, or important markers to determine susceptibility and risk of diseases such as Crohn's disease 26 and interstitial lung disease (ILD) 27. The rs3761548 was selected because it was suggested that the* FOXP3* rs3761548 SNPs could affect the susceptibility to gastric adenocarcinoma (GA) and the serum IL-35, IL-10, and TGF-β concentrations 20, and the rs3761548 polymorphism of *FOXP3* gene may provide as a risk factor in the development of breast cancer (BC) 28. The rs3761549 was selected because the rs3761549 variants may play a role to protect against high-risk human papilloma virus (HR-HPV) and cervical cancer malignant lesions by down-regulating FOXP3 18.

### *FOXP3* SNPs genotyping

Assessment of allelic discrimination for the *FOXP3* rs3761547, rs3761548, rs3761549, and rs2232365 SNP was performed with an ABI StepOne Software v2.3 Real-Time PCR System. The TaqMan assay was applied for the analysis of genotyping. The analysis and calculation of the collected data of genotyping was processed with the SDS 7000 series software (Applied Biosystems, Foster City, CA, USA) 29.

### Statistical analysis

To compare the age (years), betel quid chewing, cigarette smoking, alcohol drinking, tumor stage, tumor T status, lymph node status, metastasis, and cell differentiation, the student's t test or Chi-squared test was performed between the patients with oral cancer and the controls. A p < 0.05 was suggested to present statistically significant. To compare the odds ratio (OR) with their 95% confidence intervals (CIs) of the association between the oral cancer risk and genotypic frequencies, and the clinical pathological statuses, the data was assessed and analyzed by multiple logistic regression models. All the data analysis in our study was evaluated with SAS statistical software (Version 9.1, 2005; SAS Institute, Cary, NC).

## Results

The distribution of demographical characteristics in 1053 controls and 1175 male patients with oral cancer was listed in Table 1. In the current study, we observed that the distributions of age (years) ≦ 55 was 531 (50.4%) in controls and 601 (51.2%) in oral cancer patients. The distributions of environmental risk factors exposure between the controls and oral cancer patients were 175 (16.6%) and 856 (72.9%) in betel quid chewing (p < 0.001), 559 (53.1%) and 985 (83.8%) in cigarette smoking (p < 0.001), and 213 (20.2%) and 536 (45.6%) in alcohol drinking (p < 0.001), respectively.

The genotype distributions of *FOXP3* gene polymorphisms in 1053 controls and 1175 male patients with oral cancer were listed in Table 2. The highest distribution frequencies in patients with oral cancer of *FOXP3* genetic polymorphisms rs3761547, rs3761548, rs3761549, and rs2232365 were polymorphic variant T, polymorphic variant G, polymorphic variant G, and polymorphic variant T, respectively. The odds ratios (ORs) with their 95% confidence intervals (CIs) were estimated by logistic regression models. After adjustment for the effects of age, betel quid chewing, cigarette smoking, and alcohol drinking, no significant associations were found between the oral cancer patients and the controls (Table 2).

We further analyzed the ORs and 95% CIs of oral cancer associated with *FOXP3* genotypic frequencies among betel quid chewer. In 142 of total 856 patients, a significant association was found in those individuals who carried the *FOXP3* rs3761548 polymorphic variant T, with a lower risk of oral cancer susceptibility [The adjusted odds ratio (AOR) (95% CI):0.649 (0.437-0.964); p = 0.032] (Table 3). Moreover, after we analyzed the AOR and 95% CI of clinical statuses associated with genotypic frequencies of *FOXP3* rs3761548 in oral cancer patients, we found that in 856 betel quid chewers among the total 1175 oral cancer patients, carriers who with the rs3761548 polymorphic “T” variant have a lower risk to develop poorer cell differentiated grade [(AOR) (95% CI):0.592 (0.377-0.930); p = 0.023] (Table 4). Besides, in 536 alcohol drinkers among the total 1175 oral cancer patients, carriers who with the rs3761548 genotypic “T” variant have a lower risk to develop greater tumor size [(AOR) (95% CI):0.609 (0.378-0.983); p = 0.042] and poorer cell differentiated grade [(AOR) (95% CI):0.440 (0.248-0.779); p = 0.005] (Table 5).

## Discussion

In this study, we revealed the associations between the *FOXP3* SNPs and oral cancer. The betel quid chewing, smoking, and alcohol consumption are well-known risk factors responsible for impaired DNA repair capacity in OSCC carcinogenesis, disease development, and progression 30-34. Most of the oral cancer patients in Taiwan were male who exhibit habits of cigarette smoking as well as betel nut chewing, and over 90% of oral cancer was OSCC 35. In our study, statistically significant associations of these risk factors were found in 1175 male patients with oral cancer compared with the controls, respectively (p < 0.001, table 1).

For the correlations and hazard of these risk factors of OSCC to FOXP3 expression, a previous study of pediatric asthma has suggested that environmental tobacco smoke (ETS) exposure and passive smoking could induce pediatric asthma by affecting the balance of Treg/Th17 cells, and passive smoking was associated with significantly reduced levels of FOXP3 and tumor growth factor-β, which were associated with Treg cells 36. For the effect of alcohol exposure and FOXP3 expression, it was suggested that the alcohol interferes with the kinetics of FOXP3, and influences the balance of Treg/Th17 cells following lipopolysaccharide (LPS) exposure 37. In Taiwan, in most cases, more than 80% of the male oral cancer patients were both smokers and betel quid chewers 38. Previous study has observed that it took two decades on average of betel quid chewing and cigarette smoking before oral cavity cancer diagnosis, and the amount of life-time consumption for these substances was astonishing to these patients 38, suggesting a potentially great impact of these carcinogenic substances to FOXP3 expression in these patients. However, the associations of betel quid chewing to FOXP3 expression remained unclear till date.

We further examined the influence of the *FOXP3* genotypic frequencies to oral cancer susceptibility. Previous studies have suggested the controversial role and inconsistency of* FOXP3* gene polymorphisms to disease susceptibility in various cancers 21, 39-41. In our study, no statistical significant associations were found between the oral cancer patients and the controls, suggesting a limited carcinogenic effect and disease susceptibility of *FOXP3* polymorphisms to oral cancer (Table 2). Intriguingly, after we analyzed the *FOXP3* genotypic frequencies among betel chewers in our study, a statistical significant association was found between the oral cancer patients and the normal controls that carried the genotypic variant “T” of *FOXP3* rs3761548, with a lower risk to develop oral cancer [AOR (95% CI) = 0.649 (0.437-0.964), p = 0.032] (Table 3)*.* Moreover, after we analyzed the clinical statuses associated with genotypic frequencies of *FOXP3* rs3761548 in oral cancer patients, we found that those betel quid chewers among the oral cancer patients who carried the *FOXP3* rs3761548 polymorphic variant “T” were associated with lower risk of moderate or poor cell differentiated grade [AOR (95% CI) = 0.592 (0.377-0.930), p = 0.023] (Table 4). Furthermore, for those alcohol drinkers among the male oral cancer patients in our study, individuals who carried the genotypic variant “T” of *FOXP3* rs3761548 were found to be associated with lower risk to develop larger tumor size [AOR (95% CI) = 0.609 (0.378-0.983), p = 0.042] and moderate or poorer cell differentiated grade [AOR (95% CI) = 0.440 (0.248-0.779), p = 0.005] (Table 5). These results have suggested a possible phenomenon that the *FOXP3* SNPs (rs3761548, especially) may have greater impact and influence to oral cancer disease progression and development rather than tumor carcinogenesis (Table 3-5).

The role of FOXP3 expression in OSCC remained controversial. Some studies have associated the tumor-infiltrating FOXP3+ T cells and FOXP3 overexpression with poor prognosis in OSCC 17, 42, whereas some studies have indicated that high numbers of FOXP3+ T cells were significantly associated with prolonged overall survival 43, 44, and the levels of FOXP3+ Tregs were significantly higher in surviving oral cancer patients 43, 45. For the associations between the *FOXP3* rs3761548 polymorphisms and cancer risk, most studies have associated the A allele or the AA genotype of rs3761548 with increased cancer susceptibility, disease progression, and poor prognosis 20, 41, 46-50. However, in contrast, a previous study which focused on human papillomavirus infection and cervical cancer precursor lesions has suggested that the homozygous genotype of the rs3761548 variants (A/A) may exert a protective role against HPV infection in women, and the rs3761548 variants (A/A) was observed to be related to decreased FOXP3 expression 19. Compared with this result, in a study of Triple negative breast cancer (TNBC), it was observed that a positive association for *FOXP3* rs3761548 homozygous AA was related to TNBC susceptibility, and most of the TNBC patients (83%) have showed a strong staining for FOXP3 protein in the tumor cells, suggesting a positive association of FOXP3 expression with TNBC susceptibility and prognosis 50. One possible mechanism to explain these discriminations and inconsistencies was that it is not the overall expression of FOXP3 but its intracellular localization which really represents a prognostic parameter 51. The elevated levels of the cytoplasmicFOXP3 (cFOXP3)/nuclear FOXP3 (nFOXP3) ratio within tumor infiltrating CD4(+) T cells was proposed as a predictor of OSCC recurrence 51, and alternative splicing of FOXP3 (FOXP3Δ2, FOXP3Δ7, and FOXP3Δ2Δ7 isoforms) may play a critical mechanism which enables different aspects of FOXP3 function in T cells 52. The limitations to our study is that we lack of the data of FOXP3 expression levels and the exact cellular localization of FOXP3 to these oral cancer patients, so more detailed analysis could not be performed. Future well-designed studies are required to evaluate the mechanisms of *FOXP3* rs3761548 SNPs to oral cancer, especially the associations between the *FOXP3* SNPs to FOXP3 expression levels and their shifting balance of cytoplasmic and nuclear localization during OSCC progression, which ultimately impact and influence the oral cancer disease prognosis.

In conclusion, our study first demonstrated the associations of *FOXP3* polymorphisms to oral cancer disease susceptibility and clinical statuses. The betel quid chewers in male oral cancer patients who carried the *FOXP3* rs3761548 polymorphic variant “T” were significantly associated with lower risk to develop oral cancer and moderate or poorer cell differentiated grade. The carriers of the *FOXP3* rs3761548 polymorphic variant “T” in male oral cancer patients who with alcohol consumption were associated with lower risk to develop greater tumor size and poorer cell differentiated grade. The *FOXP3* rs3761548 may play a role as pivotal tumor marker to predict and evaluate oral cancer disease susceptibility, tumor progression, and prognosis.

## Figures and Tables

**Table 1:** The distributions of demographical characteristics in 1053 controls and 1175 male patients with oral cancer

Variable	Controls (N=1053)	Patients (N=1175)	p value
Age (yrs)			
≦ 55	531 (50.4%)	601 (51.2%)	p = 0.734
> 55	522 (49.6%)	574 (48.8%)	
Betel quid chewing			
No	878 (83.4%)	319 (27.1%)	
Yes	175 (16.6%)	856 (72.9%)	p < 0.001*
Cigarette smoking			
No	494 (46.9%)	190 (16.2%)	
Yes	559 (53.1%)	985 (83.8%)	p < 0.001*
Alcohol drinking			
No	840 (79.8%)	639 (54.4%)	
Yes	213 (20.2%)	536 (45.6%)	p < 0.001*
Stage			
I+II		554 (47.2%)	
III+IV		621 (52.8%)	
Tumor T status			
T1+T2		584 (49.7%)	
T3+T4		591 (50.3%)	
Lymph node status			
N0		781 (66.5%)	
N1+N2+N3		394 (33.5%)	
Metastasis			
M0		1165 (99.2%)	
M1		10 (0.8%)	
Cell differentiation			
Well differentiated		169 (14.4%)	
Moderately or poorly differentiated		1006 (85.6%)	

Mann-Whitney U test or Fisher's exact test was used between healthy controls and patients with oral cancer. * p value < 0.05 as statistically significant.

**Table 2:** Odds ratio (OR) and 95% confidence interval (CI) of oral cancer associated with *FOXP3* genotypic frequencies.

Variable	Controls (N=1053) (%)	Patients (N=1175) (%)	OR (95% CI)	AOR (95% CI)
**rs3761547**				
T	825 (78.4%)	930 (79.2%)	1.000 (reference)	1.000 (reference)
C	228 (21.6%)	245 (20.8%)	0.953 (0.778-1.168)	0.902 (0.703-1.157)
**rs3761548**				
G	845 (80.2%)	965 (82.1%)	1.000 (reference)	1.000 (reference)
T	208 (19.8%)	210 (17.9%)	0.884 (0.714-1.094)	0.925 (0.713-1.200)
**rs3761549**				
G	851 (80.8%)	920 (78.3%)	1.000 (reference)	1.000 (reference)
A	202 (19.2%)	255 (21.7%)	1.168 (0.950-1.436)	1.118 (0.868-1.439)
**rs2232365**				
T	654 (62.1%)	718 (61.1%)	1.000 (reference)	1.000 (reference)
C	399 (37.9%)	457 (38.9%)	1.043 (0.879-1.238)	1.058 (0.859-1.305)

The odds ratio (OR) with their 95% confidence intervals were estimated by logistic regression models. The adjusted odds ratio (AOR) with their 95% confidence intervals were estimated by multiple logistic regression models after controlling for age, betel quid chewing, cigarette smoking, and alcohol drinking.

**Table 3:** Odds ratio (OR) and 95% confidence interval (CI) of oral cancer associated with *FOXP3* genotypic frequencies among betel quid chewer.

Variable	Controls (N=176) (%)	Patients (N=856) (%)	OR (95% CI)	AOR (95% CI)^a^
**rs3761547**				
T	132 (75.4%)	674 (78.7%)	1.000 (reference)	1.000 (reference)
C	43 (24.6%)	182 (21.3%)	0.829 (0.566-1.213)	0.845 (0.576-1.239)
**rs3761548**				
G	134 (76.6%)	714 (83.4%)	1.000 (reference)	1.000 (reference)
T	41 (23.4%)	142 (16.6%)	**0.650 (0.439-0.963)^b^**	**0.649 (0.437-0.964)^c^**
**rs3761549**				
G	140 (80.0%)	665 (77.7%)	1.000 (reference)	1.000 (reference)
A	35 (20.0%)	191 (22.3%)	1.149 (0.767-1.720)	1.161 (0.774-1.742)
**rs2232365**				
T	101 (57.7%)	530 (61.9%)	1.000 (reference)	1.000 (reference)
C	74 (42.3%)	326 (38.1%)	0.840 (0.603-1.168)	0.855 (0.613-1.191)

The odds ratio (OR) with their 95% confidence intervals were estimated by logistic regression models. ^a^ The adjusted odds ratio (AOR) with their 95% confidence intervals were estimated by multiple logistic regression models after controlling for age, cigarette smoking, and alcohol drinking. ^b^ p = 0.032;^c^ p = 0.032.

**Table 4:** Odds ratio (OR) and 95% confidence intervals (CI) of clinical statuses associated with genotypic frequencies of *FOXP3* rs3761548 in male oral cancer patients.

	Total (N=1175)			Betel Quid Chewers (N=856)			Non-Betel Quid Chewers (N=319)		
Variable	G (N=965)	T (N=210)	p value	G (N=714)	T (N=142)	p value	G (N=251)	T (N=68)	p value
**Clinical Stage**									
Stage I+II	453 (46.9%)	101 (48.1%)	0.794	336 (47.1%)	71 (50.0%)	0.536	117 (46.6%)	30 (44.1%)	0.544
Stage III+IV	512 (53.1%)	109 (51.9%)		378 (52.9%)	71 (50.0%)		134 (53.4%)	38 (55.9%)	
**Tumor size**									
≦ T2	475 (49.2%)	109 (51.9%)	0.432	355 (49.7%)	76 (53.5%)	0.402	120 (47.8%)	33 (48.5%)	0.943
> T2	490 (50.8%)	101 (48.1%)		359 (50.3%)	66 (46.5%)		131 (52.2%)	35 (51.5%)	
**Lymph node metastasis**									
No	637 (66.0%)	144 (68.6%)	0.499	478 (67.0%)	100 (70.4%)	0.434	159 (63.4%)	44 (64.7%)	0.949
Yes	328 (34.0%)	66 (31.4%)		236 (33.0%)	42 (29.6%)		92 (36.6%)	24 (35.3%)	
**Metastasis**									
M0	958 (99.3%)	207 (98.6%)	0.311	710 (99.4%)	139 (97.9%)	0.087	248 (98.8%)	68 (100.0%)	-
M1	7 (0.7%)	3 (1.4%)		4 (0.6%)	3 (2.1%)		3 (1.2%)	0 (0.0%)	
**Cell differentiated grade**									
Well	133 (13.8%)	36 (17.1%)	0.193	101 (14.1%)	31 (21.8%)	**0.023^a^**	32 (12.8%)	5 (7.4%)	0.143
Moderate or poor	832 (86.2%)	174 (82.9%)		613 (85.9%)	111 (78.2%)		219 (87.2%)	63 (92.6%)	

Cell differentiate grade: grade I: well differentiated; grade II: moderately differentiated; grade III: poorly differentiated. ^a^ The adjusted odds ratio (AOR) with their 95% confidence intervals were estimated by multiple logistic regression models after controlling for age, cigarette smoking, and alcohol drinking. AOR = 0.592 (0.377-0.930).

**Table 5:** Odds ratio (OR) and 95% confidence intervals (CI) of clinical statuses associated with genotypic frequencies of *FOXP3* rs3761548 in male oral cancer patients with or without alcohol consumption.

	Alcohol Drinkers (N=536)				Non-Alcohol Drinkers (N=639)			
Variable	G (N=451)	T (N=85)	AOR (95% CI)^a^	p value^a^	G (N=514)	T (N=125)	AOR (95% CI)	p value
**Clinical Stage**								
Stage I+II	204 (45.2%)	44 (51.8%)	1.000 (reference)	0.300	249 (48.4%)	57 (45.6%)	1.000 (reference)	0.527
Stage III+IV	247 (54.8%)	41 (48.2%)	0.781 (0.490-1.245)		265 (51.6%)	68 (54.4%)	1.136 (0.765-1.689)	
**Tumor size**								
≦ T2	227 (50.3%)	53 (62.4%)	1.000 (reference)	**0.042**	248 (48.3%)	56 (44.8%)	1.000 (reference)	0.454
> T2	224 (49.7%)	32 (37.6%)	**0.609 (0.378-0.983)**		266 (51.7%)	69 (55.2%)	1.163 (0.783-1.729)	
**Lymph node metastasis**								
No	287 (63.6%)	57 (67.1%)	1.000 (reference)	0.597	350 (68.1%)	87 (69.6%)	1.000 (reference)	0.695
Yes	164 (36.4%)	28 (32.9%)	0.875 (0.532-1.437)		164 (31.9%)	38 (30.4%)	0.918 (0.598-1.408)	
**Metastasis**								
M0	446 (98.9%)	83 (97.7%)	1.000 (reference)	0.325	512 (99.6%)	124 (99.2%)	1.000 (reference)	0.624
M1	5 (1.1%)	2 (2.3%)	2.312 (0.436-12.261)		2 (0.4%)	1 (0.8%)	1.842 (0.160-21.213)	
**Cell differentiated grade**								
Well	56 (12.4%)	21 (24.7%)	1.000 (reference)	**0.005**	77 (15.0%)	15 (12.0%)	1.000 (reference)	0.378
Moderate or poor	395 (87.6%)	64 (75.3%)	**0.440 (0.248-0.779)**		437 (85.0%)	110 (88.0%)	1.308 (0.721-2.372)	

Cell differentiate grade: grade I: well differentiated; grade II: moderately differentiated; grade III: poorly differentiated. ^a^ The adjusted odds ratio (AOR) with their 95% confidence intervals were estimated by multiple logistic regression models after controlling for age, cigarette smoking, and betel quid chewing.
